# Highly divergent ancient gene families in metagenomic samples are compatible with additional divisions of life

**DOI:** 10.1186/s13062-015-0092-3

**Published:** 2015-10-26

**Authors:** Philippe Lopez, Sébastien Halary, Eric Bapteste

**Affiliations:** Team ‘Adaptation, Integration, Reticulation, Evolution’ – UMR CNRS 7138 Evolution Paris Seine – Institut de Biologie Paris Seine – Université Pierre et Marie Curie, 7 quai St Bernard, 75005 Paris, France; Département de Sciences Biologiques, Institut de recherche en biologie végétale, Université de Montréal, Montréal, QC H1X 2B2 Canada

**Keywords:** Microbiology, Metagenomics, Comparative analysis, Networks, Environmental diversity

## Abstract

**Background:**

Microbial genetic diversity is often investigated via the comparison of relatively similar 16S molecules through multiple alignments between reference sequences and novel environmental samples using phylogenetic trees, direct BLAST matches, or phylotypes counts. However, are we missing novel lineages in the microbial dark universe by relying on standard phylogenetic and BLAST methods? If so, how can we probe that universe using alternative approaches? We performed a novel type of multi-marker analysis of genetic diversity exploiting the topology of inclusive sequence similarity networks.

**Results:**

Our protocol identified 86 ancient gene families, well distributed and rarely transferred across the 3 domains of life, and retrieved their environmental homologs among 10 million predicted ORFs from human gut samples and other metagenomic projects. Numerous highly divergent environmental homologs were observed in gut samples, although the most divergent genes were over-represented in non-gut environments. In our networks, most divergent environmental genes grouped exclusively with uncultured relatives, in maximal cliques. Sequences within these groups were under strong purifying selection and presented a range of genetic variation comparable to that of a prokaryotic domain.

**Conclusions:**

Many genes families included environmental homologs that were highly divergent from cultured homologs: in 79 gene families (including 18 ribosomal proteins), Bacteria and Archaea were less divergent than some groups of environmental sequences were to any cultured or viral homologs. Moreover, some groups of environmental homologs branched very deeply in phylogenetic trees of life, when they were not too divergent to be aligned. These results underline how limited our understanding of the most diverse elements of the microbial world remains, and encourage a deeper exploration of natural communities and their genetic resources, hinting at the possibility that still unknown yet major divisions of life have yet to be discovered.

**Reviewers:**

This article was reviewed by Eugene Koonin, William Martin and James McInerney.

**Electronic supplementary material:**

The online version of this article (doi:10.1186/s13062-015-0092-3) contains supplementary material, which is available to authorized users.

## Open peer review

Reviewed by Eugene Koonin, William Martin and James McInerney. For the full reviews, please go to the Reviewers’ comments section.

## Background

The study of environmental sequences has repeatedly shown evidence for novel divisions of cellular lineages. In particular, the number of bacterial and archaeal lineages in the ribosomal tree has continued growing since Woese published a first tree of life in 1987 [1, 2]. Importantly, these environmental sequences are rarely identical to sequences from cultured organisms, which are estimated to represent less than 1 % of species diversity [3]. Many environmental sequences resemble other environmental sequences, and have no hits in homology searches outside the sequences from their own metagenome [4, 5]. These observations suggest that phylogenetic diversity inferences based on cultivation studies are still limited [1, 6], and that a vast “microbiological dark matter” still deserves to be critically analyzed [7, 8].

There has been constant progress on that front. In 1998, new division-level bacterial lineages were reported in a Yellowstone hot spring, the Obsidian Pool [1]. Evidence was obtained from small subunit rRNA genes by restriction fragment length polymorphism. Thirty percent of the genes were unaffiliated with recognized bacterial divisions, amounting to 12 novel candidate divisions. The majority of the environmental sequences produced in that study were only modestly related to known ribosomal sequences (showing less than 85 % identity with known sequences). Two of these novel divisions presented more than two representatives, giving a better sense of the significant phylogenetic depth of these lineages. Likewise, in 2005, two novel bacterial divisions were described in an anaerobic wastewater plant[2]. Again, evidence came from the analysis of 16S rRNA genes. At that time already, a third of the known bacterial divisions had no cultured representatives and were exclusively known through their detection via ribosomal sequences and universal primers [2]. Recently Brown and Luef reported the existence of CPR, a basal group comprising over 15 % of the bacterial domain and resisting cultivation because of their extremely small size, limited number of ribosomes, and their metabolic dependence coupled with the presence of pilus-like structures enabling compensatory interactions [6, 9]. Moreover the bacterial domain was not the only one in which repeated findings of novel divergent groups occurred (see also [10–15]). Phylogenetic analyses of environmental sequences also hinted at the existence of previously unobserved archaea [16–18] and eukaryotic lineages [19–22].

The growing use of next-generation sequencing further enhanced the depth of the environmental sampling, amplifying this general trend of discovery of novel lineages. As a result, uncultivated microbes detected in SSU rRNA surveys have quadrupled since 2007, reaching > 85 % of the known microbial diversity reported to date [23]. Lately, this pattern of recurrent discovery of novel microbial diversity has even suggested the existence of novel higher level groups, such as novel candidate domains of life. The key observation for this was the discovery of environmental sequences appearing highly distantly related to sequences from cultured organisms, as introduced in the pioneering work by Wu *et al.* [24].

All these results prompted the search for novel reference genomes outside the usual cultured prokaryotic and viral phyla [6, 9, 23, 25]. In 2012, the genomes of two unusual ultrasmall free-living uncultivated Archaea from a hypersaline Australian lake were *de novo* assembled [26]. These environmental taxa presented distinctive metabolic features and amino-acid composition with respect to other archaea, and 60 % of their predicted proteins could not be phylogenetically assigned. Their 16S rRNA was only 68 % to 75 % similar to cultured representatives of haloarchaea. Phylogenetic analyses of multiple markers managed to position them as members of a new major class, called Nanohaloarchaea. Similarly, in 2013, two giant viruses, *Pandoravirus salinus* and *Pandoravirus dulcis,* were discovered off the coast of central Chile and in a freshwater pond near Melbourne[25]. These enormous viruses proved highly unusual: only 7 % of their genes showed homology to known sequences (with an average of only 38 % similarity). These genomes were also completely devoid of capsid proteins. Evidence that unknown life forms populated the microbial world, i.e. microbes still unsequenced to date, including possibly some microbial dark matter (e.g. microbes that are especially difficult to grow in lab cultures) was ever growing.

Interestingly, just like many other environments, the human microbiome also presented an exciting share of unknown organisms [27, 28]. Comparing predicted genes from over 1 million shotgun reads to an extended reference database, Kurokawa et al. found that the sequences from the majority of gut microbes were highly divergent from previously known sequences[29]. Most of the organisms represented by these sequences were uncharacterized at the genus level. In 2010, Qin et al. investigated the diversity of fecal samples of 124 European adults and reported that only 31–48.8 % of the reads from their studies (plus those from two former studies with comparable methodology but smaller sampling size) could be assigned to 194 public human gut bacterial genomes, while 7.6-21.2 % could be assigned to the bacterial genomes from Genbank [30]. Numerous completely novel gene families without known functions, amounting together to three quarters of the gene families of the gut microbiome, were also reported [30]. One year later, Arumugam et al. analyzed metagenomes from 39 individuals and estimated that around 16.5 % of the reads were likely to belong to yet unknown genera [31]. Finally, in 2012, the use of 212 different culture conditions revealed novel species and genera in the gut microbiome, including the largest bacterial genome and the largest virus reported in that environment, demonstrating that metagenomic studies still underestimate microbial diversity within the gut [8]. Thus, wherever one looks in nature – from hot springs to human guts-, novel microbial lineages from various taxonomic levels are being discovered through environmental sequencing.

However, a general question remains to be addressed: are we missing novel lineages in the microbial dark universe by relying on standard phylogenetic and BLAST methods? If so, how can we probe that universe using alternative sensitive approaches? Indeed, studies of microbial dark matter using metagenomic data raise significant methodological challenges for comparative analyses. A first difficulty comes from the size of the dataset. For instance, in 2012, Lynch et al. devised a strategy to analyze 6.5 million assembled paired-end Illumina reads of 16S rRNA from Arctic tundra soil samples [32], in which they found 3 very divergent bacterial lineages. To handle this large amount of data, the authors relied first on sequence similarity networks. Nodes in these networks corresponded to abundant environmental 16S rRNA sequences and reference sequences from a database with 2,000,000 curated assigned sequences, connected by edges when they presented > 90 % identity and matched over > = 80 % of their hit length. In such networks, candidate new lineages are typically unconnected to reference sequences, because they are highly divergent [32]. This study had a strong methodological implication: novel comparative strategies and tools are critical for the screening of the microbial dark matter, because reconstructing a tree of every 16S rRNA sequences was no longer a tractable default option (or at least became a real challenge).

Comparative strategies targeting novel lineages however need to be designed with the specific goal of enhancing the exploratory power of microbial diversity analyses, which could also benefit microbial dark matter analyses. Thus, a second difficulty in analyzing microbial diversity comes from the very nature of novel lineages, which are by definition very divergent from known lineages. Indeed, divergent 16S ribosomal RNA gene sequences evade detection in typical cultivation-independent surveys [9]. Similarly, the most divergent environmental sequences can hardly be included in comparative phylogenetic analyses, because divergent environmental sequences and sequences from cultured organisms often fail to align sufficiently well when introduced together in multiple sequence alignments. Therefore, the usual phylogenetic approaches, even though they appear very promising for species assignation, might impose methodological constraints that are too restrictive to serve as first tools of choice for exploratory searches of highly divergent environmental sequences. For methodological reasons, it might be difficult, if not practically impossible, to use truly divergent genes from truly divergent life forms, such as very divergent groups or eventual additional domains of Life, to reconstruct phylogenetic trees that simultaneously contain both novel divergent lineages branching outside (rather than within) the three domains of Life and sequences from Archaea, Bacteria and Eukaryotes. Problematically, most intriguing environmental sequences might then tend to be discarded in the first steps of classic comparative analyses of genetic diversity, making it less likely that very divergent lineages could be easily detected by these approaches [9].

Consequently, to show that the microbial dark universe could be probed by alternative methods, we developed a novel protocol for the exploitation of huge numbers of molecular sequences in exploratory diversity surveys. We designed a multi-marker sampling strategy, relying on sequence similarity network to identify (i) well-distributed gene families in cultured prokaryotes and (ii) their distant environmental homologs, characterized by their topological distance to known sequences in these graphs and their significant sequence divergence (>40 %) from their closest published homolog present in the NCBI database in July 2013. We applied these tools to environmental samples, with a particular focus on the human gut microbiome, because these data are amongst the most abundant and cleanest (and therefore most convenient) publicly available, and because establishing the gut microbiome composition is one of the major challenges of the 21st century.

## Results

We designed an analysis to demonstrate that divergent environmental sequences can be investigated fruitfully using networks. Starting with 571,043 sequences from 54 archaeal, 70 bacterial and 7 eukaryotic complete genomes, we first defined 40,584 gene families by clustering sequences with > = 30 % identity over > = 80 % of their lengths as in [32]. We then selected gene families for which finding divergent environmental homologs would be of utmost interest. To do so, we sorted these gene families based on taxonomical and topological criteria in sequence similarity graphs to identify gene families with at least two densely connected modules of sequences, each from a distinct domain of life. In other words, we sought to identify ancient gene families that show a strong divergence between the two prokaryotic domains, are rarely transferred between archaea and bacteria, and are widely distributed over each domain. In terms of networks (see Methods), this objective translated into selecting connected components with a conductance of < 0.4 for each prokaryotic domain. This conservative threshold yielded only 86 gene families (0.2 %) fulfilling these conditions. These gene families are both broadly distributed across cellular life and show no sign of inter-domain gene transfer, i.e. bacterial sequences formed a group in the graph that was distinct from the group of archaeal sequences. When homologs were present in the 3 domains of life, our graph presented the typical pattern described in [33], with eukaryotic sequences from bacterial origins connecting to bacterial sequences while eukaryotic sequences from archaeal origins connected to archaeal sequences. Bacterial and archaeal homologs within these well-distributed, rarely transferred between domains, gene families showed > 60 % identity on average. This means that our protocol was not restricted to the search for members of highly conserved (in terms of primary sequences) families. All gene families with a strong signal in the network could be exploited. These sequence showed clear homology as well as true divergence between archaeal and bacterial sequences and allowed us to depart from the conventional set of highly conserved markers, such as those found by AMPHORA [34] or PhyEco [35], and hence to offer a complementary analysis of genetic diversity. Given that archaeal and bacterial homologs shared at least 60 % sequence identity, any environmental homologs of these gene families presenting > 40 % divergence (i.e., <60 % identity) would be more divergent from its homologs than sequences from two distinct domains of life. Such a high divergence, for these families, deserves to be considered significant, possibly hinting at very divergent organismal lineages, and/or reflecting a major genetic plasticity for these functionally important, apparently ancient gene families. It is noteworthy that the gene families meeting this criterion included genes coding for ribosomal proteins (Table 1).

**Table 1:** Main annotated functions of the gene families for which environmental homologs were identified

Main annotated functions	Samples with (#) env. homologs
maltose ABC transporter, periplasmic maltose-binding protein	HMQ(127); JM(6)
rhomboid family protein	HMQ(907); AA(95); HA(33); JM(29); BB(18); WL(6)
phage shock protein A, PspA	HMQ(111); JM(10); BB(3); AA(3)
segregation and condensation protein B	HMQ(1083); HA(120); AA(117); JM(39); BB(30); WL(11)
V-type ATP synthase subunit I	HMQ(326); AA(16); JM(15)
protein of unknown function DUF192	HMQ(94); AA(33); HA(23); BB(19); WL(10)
nucleotide kinase	HMQ(9); HA(3)
30S ribosomal protein S8e	HMQ(7); AA(5)
homoserine kinase	HMQ(274); AA(77); HA(43); JM(17); BB(8)
DNA-directed RNA polymerase subunit L	HMQ(4); HA(3)
30S ribosomal protein S24e	HMQ(6)
ribosomal biogenesis GTPase	HMQ(980); JM(32); HA(23); AA(5); WL(4); BB(3)
DNA-directed RNA polymerase I, II, and III, 7.3 kDa polypeptide	HMQ(291); AA(34); WL(5); JM(5)
protein of unknown function DUF167	HMQ(94); AA(62); WL(18); JM(3)
CoA-substrate-specific enzyme activase	HMQ(919); JM(64); AA(9); WL(4)
RNA-binding protein	HMQ(717); JM(52); WL(12); HA(10); BB(8); AA(8)
hypothetical protein	HA(43); AA(29); BB(16) HMQ(9)
protein of unknown function DUF420	AA(18); BB(8); WL(3)
twin arginine-targeting protein translocase	HMQ(344); HA(105); AA(105); BB(31); WL(20); JM(17); MB(8); SI(5); G(3)
protein of unknown function DUF502	HA(87); AA(59) HMQ(47); BB(24); WL(15)
ribonuclease III	HMQ(1370); AA(549); HA(115); JM(66); BB(51); WL(6)
polyprenyltransferase	HMQ(44); AA(44); HA(32); BB(8); WL(7)
Rossmann fold nucleotide-binding protein	HMQ(1133); AA(203); HA(79); JM(46); BB(28); WL(18)
glutamyl-tRNA reductase	HMQ(299); HA(31); AA(31); BB(14); WL(7); JM(7)
50S ribosomal protein L30P	AA(13) HMQ(12); HA(3)
50S ribosomal protein L34e	HMQ(1); HA(1); AA(2)
50S ribosomal protein L14e	AA(7)
Pre-mRNA processing ribonucleoprotein, binding region	HMQ(6)
like-Sm ribonucleoprotein, core	AA(23) HMQ(7); HA(4); BB(3)
30S ribosomal protein S26e	HA(2)
30S ribosomal protein S3Ae	HMQ(5)
30S ribosomal protein S27e	AA(8) HMQ(4)
cobalamin 5'-phosphate synthase	HMQ(871); JM(30); AA(17); HA(15); WL(8); BB(3)
phenylalanyl-tRNA synthetase subunit alpha	HMQ(1698); AA(207); HA(127); JM(66); BB(48); WL(7)
cell division protein and ATP-dependent metalloprotease FtsH	HMQ(1707); HA(338); AA(335); JM(109); BB(79); WL(6)
50S ribosomal protein L1P	HMQ(1231); AA(215); HA(146); JM(65); BB(43); WL(16)
AAA family ATPase, Cell Division Cycle CDC48 subfamily protein	HMQ(10)
methionyl-tRNA synthetase	HMQ(1390); AA(211); HA(103); JM(81); BB(35); WL(7); MB(5)
50S ribosomal protein L2P	HMQ(815); AA(227); HA(147); JM(75); BB(63); WL(3)
50S ribosomal protein L22P	HMQ(1765); AA(256); HA(150); JM(88); BB(53); WL(19); MB(3); SI(3)
inositol monophosphatase	HMQ(1349); HA(342); AA(307); BB(113); JM(55); WL(15)
chaperonin GroEL	HMQ(678); AA(191); HA(128); BB(67); JM(48); WL(5)
50S ribosomal protein L13	HMQ(1827); AA(286); HA(142); BB(63); JM(53); WL(8); MB(4); SI(4)
50S ribosomal protein L5P	HMQ(2379); AA(520); HA(373); JM(150); BB(116); WL(25); MB(9)
30S ribosomal protein S12	HMQ(956); AA(239); HA(155); JM(61); BB(57); SI(20); WL(14); MB(5); G(3)
Bcr/CflA subfamily drug resistance transporter	HMQ(399); JM(19); WL(3)
DNA repair and recombination protein RadA	HMQ(3327); AA(372); HA(195); JM(109); BB(86); WL(21)
50S ribosomal protein L6	HMQ(1402); AA(239); HA(142); JM(73); BB(55); WL(16)
methionine aminopeptidase	HMQ(6396); AA(538); HA(316); JM(217); BB(184); WL(22); LM(3)
ribonuclease HII	HMQ(2476); AA(173); HA(130); JM(60); BB(43); WL(13)
50S ribosomal protein L23	HMQ(2229); AA(253); HA(158); JM(99); BB(46); WL(22); MB(14)
molybdenum cofactor biosynthesis protein A	HMQ(2024); AA(146); JM(62); HA(49); BB(40); WL(17)
50S ribosomal protein L3P	HMQ(1404); AA(205); HA(103); BB(61); JM(61); WL(8); SI(3)
50S ribosomal protein L18e	HMQ(10); AA(5); BB(3); HA(3)
tryptophanyl-tRNA synthetase	HMQ(1877); AA(213); HA(151); JM(52); BB(45); WL(9)
aspartyl-tRNA synthetase	HMQ(3159); AA(348); HA(225); JM(149); BB(95); WL(13)
nicotinate nucleotide adenylyltransferase	HMQ(2308); AA(170); HA(138); JM(73); BB(62); WL(15)
30S ribosomal protein S17P	HMQ(1771); AA(264); HA(137); JM(79); BB(50); WL(23); MB(11); SI(10)
putative RNA methylase	HMQ(1410); HA(35); JM(34); AA(20); BB(6); WL(6)
translation-associated GTPase	HMQ(2629); AA(316); HA(247); JM(97); BB(65); WL(11)
50S ribosomal protein L29	HMQ(1969); AA(219); JM(82); HA(48); BB(24); SI(22); MB(16); WL(12)
appr-1-p processing domain-containing protein	HMQ(805); JM(55); AA(20); WL(5); BB(3)
30S ribosomal protein S7	HMQ(1095); AA(313); HA(165); BB(67); JM(67); SI(10); WL(6); G(3)
TatD-related deoxyribonuclease	HMQ(3338); AA(182); HA(128); JM(86); BB(46); WL(10) ; OM(5)
mevalonate kinase	HMQ(1131); JM(45); AA(16); HA(6)
O-sialoglycoprotein endopeptidase	HMQ(2408); AA(213); HA(163); JM(99); BB(53); WL(5); LM(3)
MiaB-like tRNA modifying enzyme	HMQ(5237); AA(252); HA(197); JM(135); BB(88); WL(12)
50S ribosomal protein L15P	HMQ(1798); AA(174); HA(133); JM(103); BB(56); WL(19); MB(4)
6,7-dimethyl-8-ribityllumazine synthase	HMQ(1365); AA(180); HA(149); JM(50); BB(38); WL(25)
30S ribosomal protein S5P	HMQ(3691); AA(416); HA(299); JM(154); BB(105); WL(23); MB(5)
carbohydrate kinase, YjeF related protein	HMQ(1547); JM(38); HA(27); AA(13); WL(8); BB(6)
ribose-phosphate pyrophosphokinase	HMQ(1807); AA(191); HA(158); JM(70); BB(39); WL(11)
replication factor C small subunit	HMQ(6239); AA(712); HA(225); JM(167); BB(86); WL(19); MB(5)
hydrolase	HMQ(6012); JM(207); HA(45); AA(22); BB(10)
tyrosyl-tRNA synthetase	HMQ(1123); AA(192); HA(128); JM(65); BB(55); WL(6)
50S ribosomal protein L18P	HMQ(14)
Fmu (Sun) domain-containing protein	HMQ(2680); JM(76); AA(50); HA(47); BB(20); WL(12)
30S ribosomal protein S2	HMQ(834); AA(225); HA(130); JM(48); BB(44); WL(8)
leucyl-(isoleucyl-) and (valyl-)tRNA synthetase	HMQ(2653); AA(112); JM(78); HA(75); BB(51); WL(7)

Environments of origins were HMQ: Human microbiome Qin2010; JM: Japanese Microbiome; AA: Antarctica Aquatic; HA: Hot Aloha; BB: Botany Bay; WL: Washington Lake; MB: Monterey Bay; SI: Sapelo Island; G: Glacier; LM: Lean Mice; OM: Obese Mice.

By contrast to direct BLAST searches [36], we used two rounds of BLAST searches against a local environmental database, comprising 9,865,367 predicted ORFs from about 33 million sequences from 236 metagenomic samples, to search for homologs of the cultured sequences of these conserved families. The first round of BLAST defined a set of environmental homologs with significant hits to known cultured sequences. In the second round, these environmental homologs were used as seeds to look for their own environmental homologs. Using this protocol we retrieved 309,245 sequences. We then applied a stringent criterion to preserve homology relationships between sequences [37], imposing a minimal alignment coverage of > = 80 % between any pair of sequences with > 30 % identity, eventually retaining 131,162 environmental sequences. We thus constructed an environmentally extended version of the network of sequences from cultured organisms, expanding its original gene families through the addition of their environmental homologs. Remarkably, almost all the graphs (82/86) of these extended gene families showed two typical patterns of connection between cultured sequences and their environmental homologs. A first class of environmental sequences was directly connected to the cultured sequences; a second class of environmental sequences was indirectly connected to the cultured sequences, being at a distance 2 (in number of edges) from these known reference genes. Indeed, class 1 environmental homologs (retrieved in the first round of BLAST search) acted as bridges to connect class 2 environmental homologs to the gene family.

**Fig. 1:**
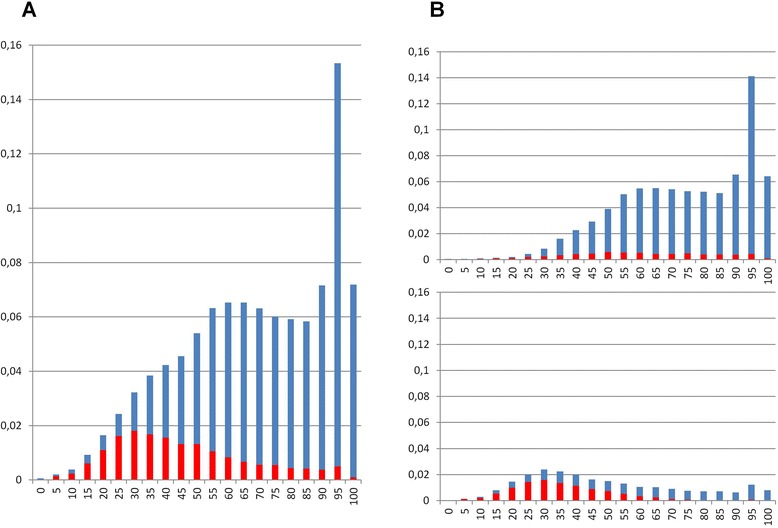
Distribution of identity percentage to closest published relative for environmental sequences. For each of the 131,162 environmental sequence retrieved by our protocol, the closest published relative is identified as the best BLAST hit against July 2013 release of *nr* NCBI database. Identity percentage between the two sequences, shown in abscissa, is computed as the hit coverage relatively to the smallest sequence multiplied by the BLAST identity percentage. The proportion of environmental sequences showing a given identity percentage is given in ordinate, in blue for sequences from human gut microbiome and in red for others. **a**) Class1 (direct link to cultivated hosts sequences) and class2 (indirect link) are cumulated. **b**) Class1 and Class2 are separated on top and bottom, respectively

All these environmental homologs were then compared against the whole NCBI *nr* database (July 2013) to look for their closest published relatives (CPR), not only in our networks, but also among all the publicly available sequences from cultured organisms and viruses, defining for each environmental sequence an identity to its CPR (Fig. 1a). 22.5 % of these environmental sequences had > =95 % identity to their CPRs. However, 44.3 % of the environmental sequences were more divergent, presenting only 60 %-95 % identity to known sequences. Finally, one third of the environmental homologs (33.2 %) showed even less similarity to CPRs than this threshold, thus being more distant from their cultured homologs than homolog pairs from different prokaryotic domains were (on average) for these gene families. This first striking result confirms that the genes in cultured organisms are not representative of 77.5 % of the genes of environmental populations. It suggests that genetic and possibly taxonomic diversity are still significantly underestimated at present. The difference between the genetic diversity of these environmental genes and that of cultured microorganisms, for these 82 important markers, raises a general problem. Inferences of microbial genetic resources and microbial taxonomic diversity made from organisms grown in Petri dishes cannot be assumed to hold as general complete descriptions. Our fundamental knowledge about the genetic and possibly the taxonomic composition of microbiomes remains biased by practical considerations. Even gene families with known functions, such as most of our well-distributed ones, have their own share of ‘genetic dark matter’. In particular, there is still a substantial number of highly divergent microbial genes in the gut. However, other environments harbor proportionally more of these divergent genes than the gut (Fig. 1a). Indeed, while non gut environmental sequences represent only 17 % of the retrieved homologs, they account for significantly larger proportions amongst the most divergent sequences, showing less than 50 % identity to CPRs (prop test p-value of 2e-16). The median of the distribution of the distance to a CPR lies around 40 % for homologs from other environments than the gut while it lies around 72 % for homologs from gut samples. This difference is likely due to the Human Microbiome Project, which has produced relatively many highly divergent sequences from gut samples. Strong sequencing efforts focusing on non-gut environments would help identify more hard-to-find, highly divergent environmental homologs. This contrasted observation *a posteriori* justifies the choice of gut samples as a major part of our dataset, and makes it obvious that massive metagenomic analyses are a relevant way to reveal more as yet unknown genetic diversity.

Importantly, the network topology provides additional insight concerning microbial genetic diversity. The distribution of identity to a CPR for class 2 environmental homologs, i.e. the sequences indirectly connected to cultured prokaryotes sequences, is significantly lower, compared to that of class 1 homologs, i.e. the sequences directly connected to cultured prokaryotic sequences (one-sided KS test, p-value 1e-3) (Fig. 1b). Consequently, sequences far away, i.e. separated by more than one edge, from cultured samples in this network are also generally more divergent. Contrary to traditional surveys of genetic diversity based on BLAST searches that only take into account direct matches, the network topology allows the detection of more distant relationships between homologs, through indirect paths of increasing length that nonetheless represent homology.

One might worry that iterative rounds of BLAST, using the last retrieved sequences as seeds, would eventually retrieve sequences unrelated to initial seeds, despite the additional criterion of 80 % mutual cover between two sequences to be considered homologs. This should not be the case if gene families under investigation occupy relatively isolated regions of the genetic space, because each new BLAST round should yield more of the genetic diversity of such gene families until exhaustion is reached. Accordingly, we observed a much lower number of class 2 than class 1 sequences. Figure 1b also shows that sequences from gut samples are found both amongst class 1 and class 2 environmental homologs, but in significantly greater proportions amongst class 1 homologs (proptest p-value of 2e-16). By contrast, sequences from other environmental samples are especially numerous in class 2 homologs. In particular, in this dataset, marine (Botany Bay), cold (Antarctica Aquatic) and hot (Hot Aloa) water environments proved very rich in these most divergent environmental homologs. This difference in relative network positions demonstrates the impact of the strong sequencing effort targeting the human gut microbiome that revealed some (but by no means all) microbial diversity in the environment.

**Fig. 2:**
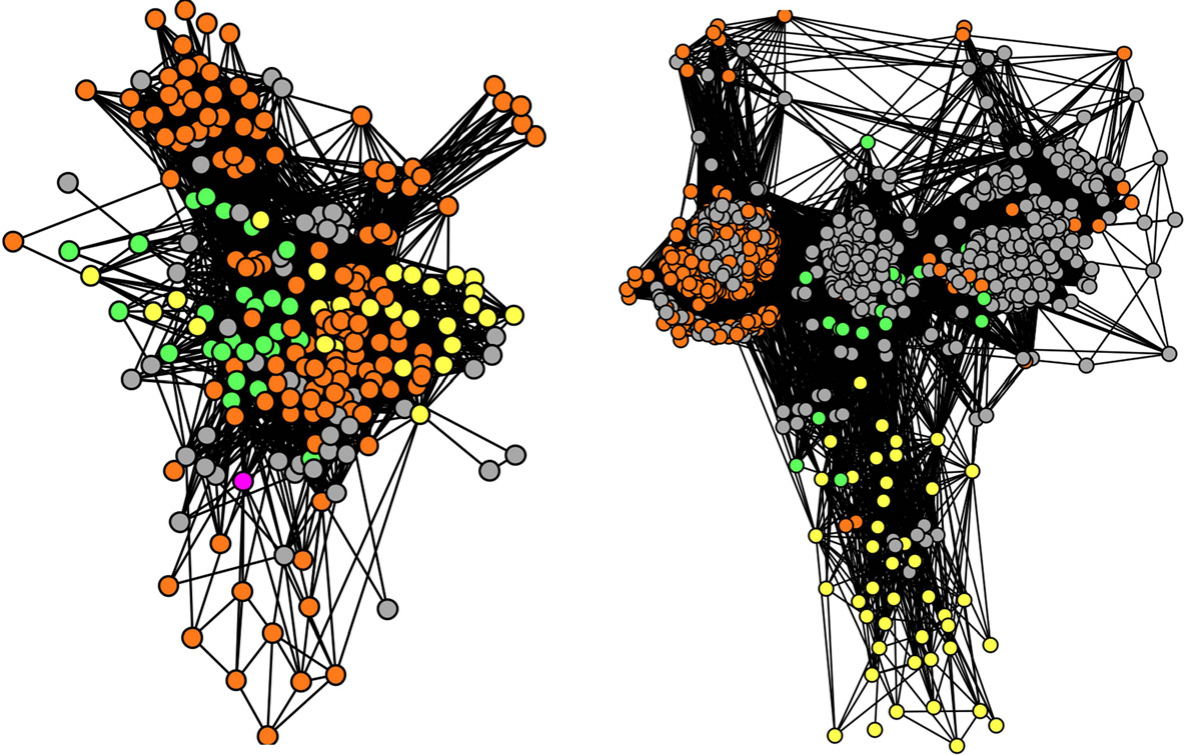
Sequence similarity networks for cultivated hosts sequences and their associated environmental sequences. Each node corresponds to a sequence. Two nodes are connected when they share > = 30 % identity, for a hit covering > = 80 % of both of their lengths, with a BLAST score < 1e-5. Sequences are yellow for Archaea, green for Bacteria, orange for environmental homologs whose identity to their closest published relative is lower than 60 %, and grey for environmental homologs whose identity to their closest published relative is higher than 60 %. Left: DUF167 protein, right: cobalamine phosphate synthase

**Fig. 3:**
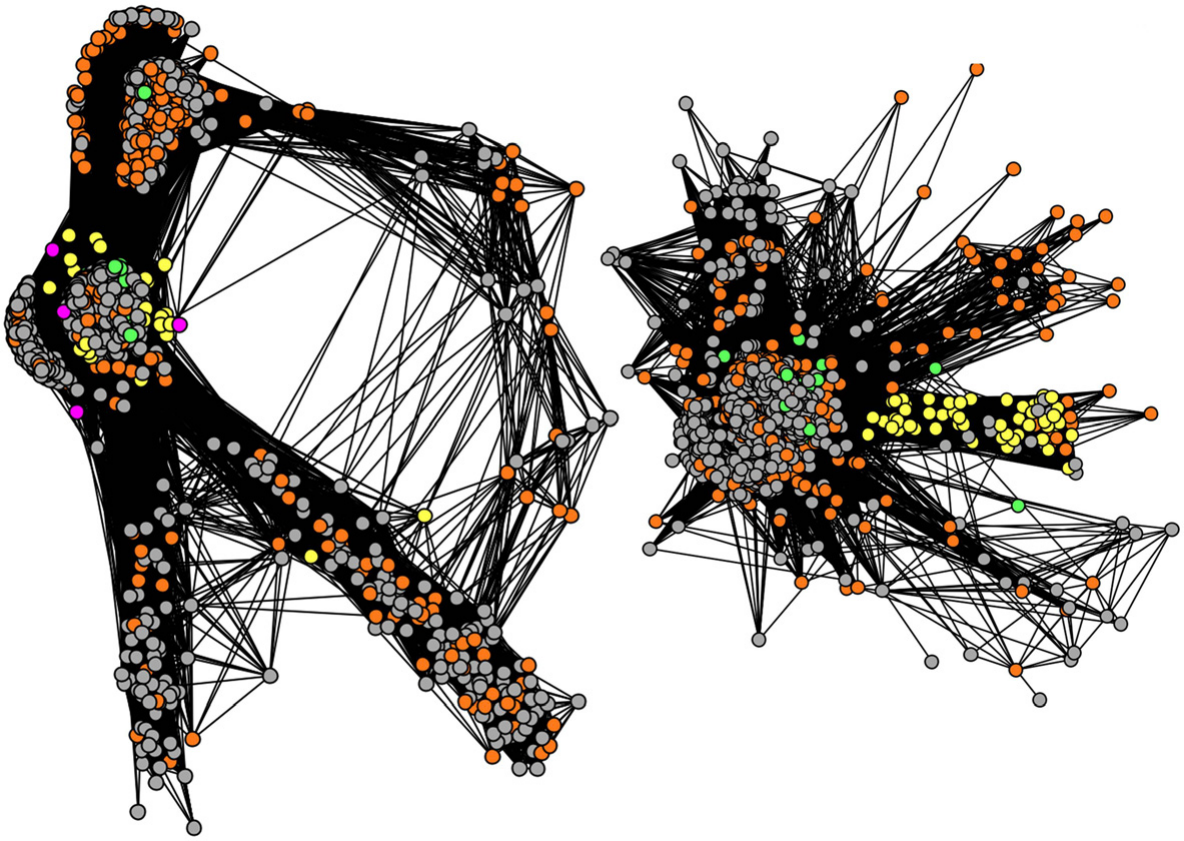
Other sequence similarity networks for cultivated hosts sequences and their associated environmental sequences. The color code is the same as in Fig 2. Left: metalloendoprotease, right: ribosomal protein RPL23/25

**Fig. 4:**
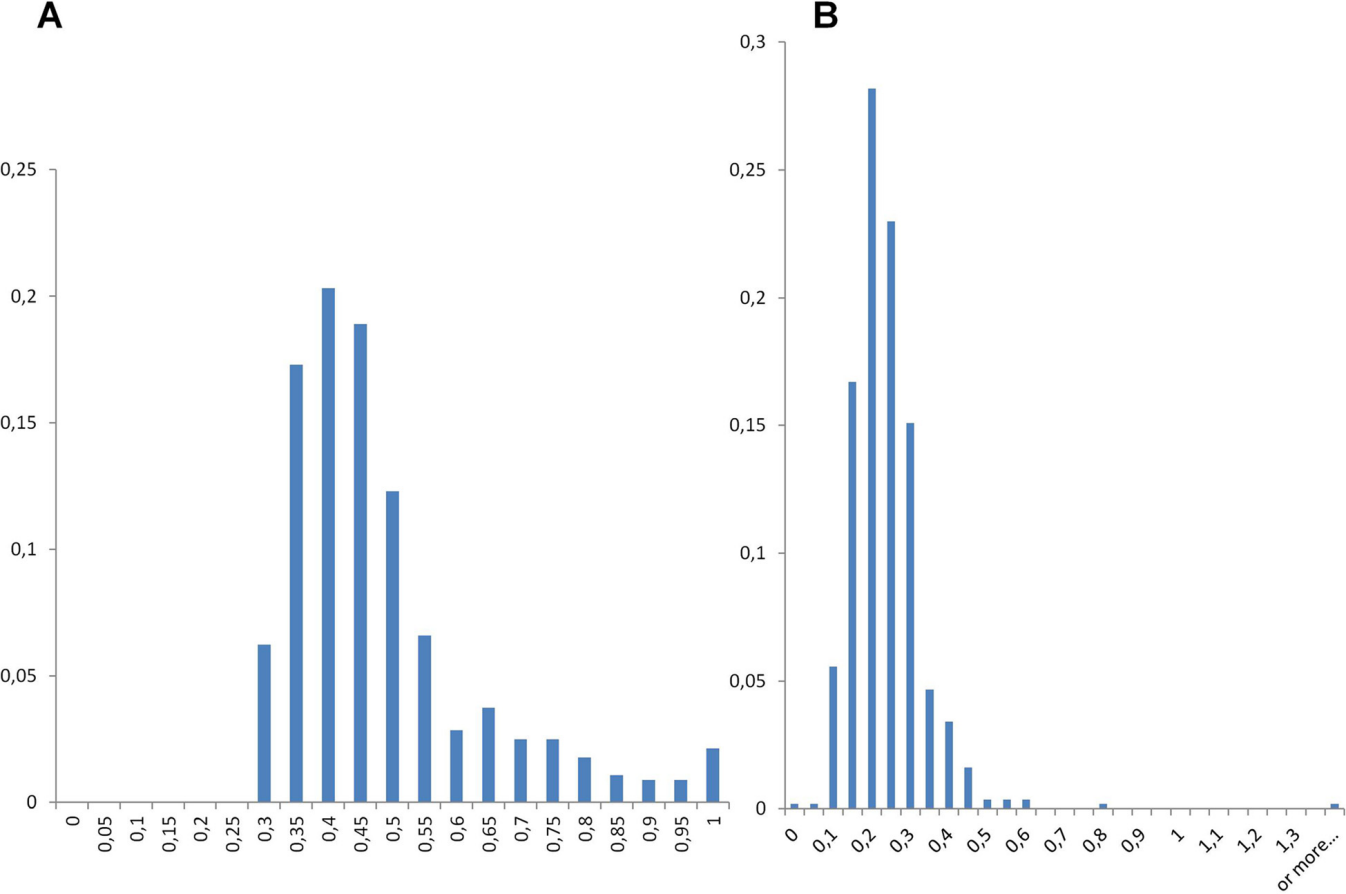
Distribution of **a**) Average Amino Acid Identity and **b**) Average dN/dS for cliques of highly divergent environmental sequences. A total of 569 cliques (totally connected subnetworks) of highly divergent environmental sequences (whose identity to CPR is lower than 60 %) was identified. Sequences from each of these cliques were aligned, and then their average amino acid identity and dN/dS were computed, and shown in abscissa. Proportion of corresponding cliques is given in ordinate

Moreover, the structure of our graphs (Figs. 2 and 3) also allowed us to distinguish groups of divergent environmental homologs, whose identity to CPRs was lower than 60 %, defining 562 tight clusters (network cliques with exclusively highly divergent environmental content) in the graphs of 69 gene families under study, including ribosomal proteins. Since identical (redundant) sequences had been preliminarily filtered out, the existence of these groups of divergent environmental homologs means that, occasionally, many related variants of a given group of divergent environmental homologs are present in nature. Importantly, these cliques should not be confused with phylotypes [38], because there was a wide range of genetic variation between environmental sequences within these groups, as measured by average nucleotide or amino acid identity (Fig. 4a and Additional file 1: Figure S1). While a few of these groups had an average identity close to 1, indicating very similar sequences, most cliques had a much lower average residue identity, confirming that the sequences therein were not just close variants of a single sequence produced by sequencing errors. In fact, the genetic variation within these groups of environmental sequences was comparable, if only slightly smaller, to that of either archaeal or bacterial cliques of the networks, a remarkable property compatible with the hypothesis that these environmental homologs belong to highly diversified taxonomical groups. Moreover, careful analyses with RDP [39] of the alignments of these related variants showed no evidence of either recombination or chimerism in these divergent environmental homologs. Furthermore, dN/dS estimates for these environmental divergent homologs revealed that most of these gene forms were under purifying selection (median of the average dN/dS distribution 0.22, Fig. 4b). These last two observations confirmed that there were very few if no confounders like PCR errors, chimeras and pseudogenes involved in this genetic diversity.

Consequently, our most comprehensive graph-based representation of genetic diversity in metagenomic samples reveals an impressive number of gene families with potentially coding, highly divergent, environmental homologs. Once again, it is worth stressing that most of this genetic diversity cannot be represented nor can it be simultaneously analyzed with their homologs from cultured organisms in conventional phylogenetic analyses, because the vast majority of our cliques of distant homologs cannot be confidently aligned with their cultured homologs. Indeed, only 289 out of 562 environmental cliques produced alignments with more than 10 unambiguously aligned positions, as assessed by GBlocks [40]. Thus, sequence similarity networks not only provide a novel, sufficient, but also a broader type of evidence for identifying clusters of highly divergent environmental sequences with respect to sequences from cultured organisms. The exploration of microbial dark matter therefore directly benefits from this alternative representation of the relationships in molecular data, and the analysis of these paths and cliques.

**Fig. 5:**
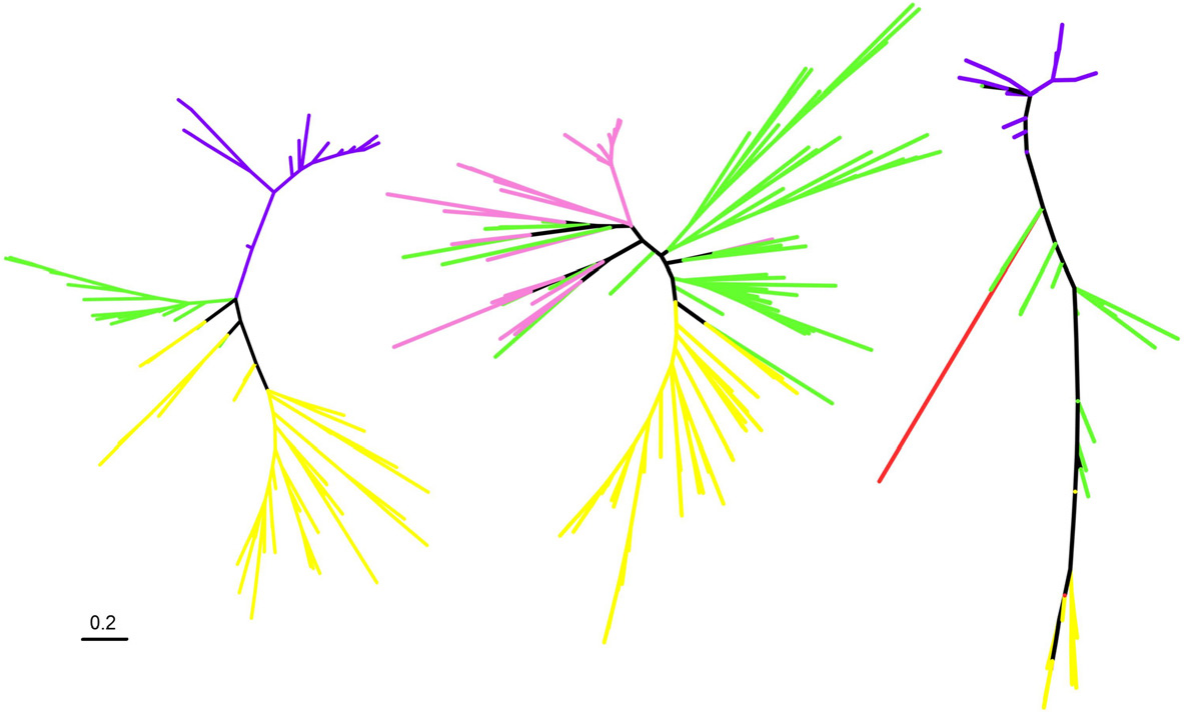
A phylogeny-based illustration of the actual divergence of some of the environmental homologs detected in our networks. Maximum likelihood phylogenetic trees were based on sequences of archaeal (yellow), bacterial (green), eukaryotes (red) and a subset of the alignable positions of some environmental homologs (purple for gut sequences, pink for other environments), extracted using a maximal clique search. Three trees presenting a remarkable pattern are shown here. Left: cobalamine phosphate synthase (27 alignable positions), middle: RPL29 (52 alignable positions), right: metalloendoprotease (10 alignable positions). A scale bar for branch lengths (number of substitutions per site) is given on bottom left

As a further indication that sequence similarity networks nonetheless also provide evidence for diversity studies consistent with that of phylogenetic trees and networks, we estimated phylogenetic trees including the minority of groups of environmental sequences that could be aligned with sequences from cultured organisms from at least 2 domains of life. These expectedly short alignments were used to reconstruct 289 maximum likelihood unrooted trees for 65 gene families. Some of these divergent environmental homologs branch within the archaeal and bacterial clans for some genes, and some also branch outside the archaeal and bacterial clans [41] (Fig. 5). Such a phylogenetic position is compatible with major deep branching divisions. Overall, this analysis further confirms the large phylogenetic depth within groups of environmental homologs detected in the network, and their significant phylogenetic distance to sequences present in cultured lineages in our dataset. Interestingly, we observed such divergent lineages of environmental genes in human microbial gut samples.

## Discussion

Our inclusive multi-marker network-based approach efficiently recovered novel evidence of highly divergent microbial genes, possibly partly originating from microbial dark matter, even in the human gut. Of course, one must be careful when interpreting this result, because the divergence between genes does not necessarily reflect the same phylogenetic divergence between organisms that carry those genes. However, although we have not been looking at 16S data, when one considers the gene families for which we report massively divergent environmental homologs under purifying selection, it must be noted that many (18) of the studied gene families with these highly divergent forms are ribosomal proteins (also commonly used in phylogenetic reconstruction [42]) and that the broad distribution and infrequent inter-domain transfer of these gene families suggests they all perform critical functions in cells. Moreover, the detection of groups of divergent environmental homologs from these families provides evidence that these divergent gene forms are not independent genome specific variants (i.e. occasional variants coming from individual, unrelated genomes) but are shared by different related genomes. That related genomes have largely diversified copies for these important gene families suggests that a significant fraction of the environmental divergent genes may come from gene families with otherwise known functions in cultured organisms. If one assumes that these variants come from known lineages, then our results suggest that functional annotation in metagenomic studies is incredibly challenging, because adaptation to different environments must lead to massive molecular changes even for such gene families. In that case, environmental divergent genes would partly result from an impressive plasticity of these genes families, affecting even ribosomal proteins, consistently with [9]. Otherwise, these genes could be the extensively diverged descendants of ancient gene duplications (with a potential history of mosaicism or gene fusion). Therefore, when our finding is cautiously interpreted at the gene level, it indicates that even gene families that are well-distributed among widely divergent lineages have undergone substantial sequence evolution in the environment.

However, the gene level is not the only possible level of explanation for the massive diversity observed in the environment. The detection of groups of environmental homologs (for which we cannot determine whether they are orthologs or paralogs, since precisely we lack their genomes of origins), more divergent on average from any known cultured or viral sequence than the observed divergence between archaea and bacteria for these gene families can also be interpreted at a higher level of biological organization. In other words, another (non-exclusive) explanation for this genetic variation might also be to consider that some novel lineages of microbes remain to be discovered. Then, the nature of the hosts of such highly divergent homologs in otherwise “cell-ubiquitous” gene families becomes a particularly intriguing question. Do divergent lineages of environmental genes point to divergent, deep-branching lineages of organismal hosts? There are good reasons to assume the present analysis, as many studies before [9], has revealed molecular cues of novel major life divisions in the environment. Of note, this seems to be the case also in the human gut. First, according to the literature, almost all the genes from these gut samples come from microbial organisms [30]. Second, since most of these genes are under purifying selection, and since they belong to relatively ubiquitous, ancient families, including ribosomal proteins, they are probably adaptive to some hosts. Thus, if taken at face value, these deep branching lineages and groups of divergent environmental genes suggest that some major organismal divisions have still not been described and are present in the human gut.

Even though environmental divergent sequences showed no match to known viral sequences, the possibility that some of these environmental divergent forms are carried by viruses rather than hosted in cells remains of course open. But invoking a viral host would either mean that viruses play the role of vehicles, mobilizing these divergent genes from unknown microbial organisms (hence signing the presence of novel organismal divisions), or that there exists a *bona fide* evolutionary connection between sequences from unknown viruses and genes from 82 ancient gene families well-distributed amongst multiple divergent cellular organisms. This connection would then implicate an ancient relationship between cell and viral lineages in the origin of these genes, as debated in [4, 43], and thus suggest a shared ancestry between large viruses and cells [44]. In either case, the host lineage of the divergent environmental genes would be distantly related to known organismal lineages in a way that may challenge our knowledge of evolutionary microbiology.

## Conclusions

Overall our protocol identifies much more divergent (environmental) gene forms than previous analyses of metagenomic data. Our approach complements and expands the scope of diversity analyses based on direct BLAST searches, trees of ribosomal genes, relatively stringent networks of ribosomal genes, or 16S phylotypes, and should prompt further sequencing of complete environmental genomes by providing primers to probe diversity for cells with highly unusual genes. More precisely, existing libraries could be probed for the hosts of these genes, using primers designed from the alignments of the highly divergent environmental genes detected in our graphs, in order to isolate and amplify their genomic material. Future analyses may also further target these hosts by cell sorting and *in situ* hybridization analyses on gut microbiome samples to provide microbiological evidence regarding the extent of the microbial dark matter within the environment and within our own body. Of course, the detection of orthologs in larger datasets using networks (currently something we have not achieved) could in principles also greatly help to this endeavour. The potential discovery of novel groups would be of major importance in microbiology and evolutionary biology. Just as the discovery of Archaea spawned an entire research field [45], one can imagine that significant progresses may also result from the discovery of major lineages and remarkable variants of important gene families within the microbial dark matter. As a follow up of this proof of concept analysis, we encourage larger comparative studies, with expanded datasets, including more sequences from cultured organisms and from more environments.

## Methods

### Constitution of the datasets

The initial dataset comprised 564,448 protein sequences from 116 prokaryotic genomes (54 Archaea and 70 Bacteria, sampled in order to cover prokaryotic diversity as in [33]), 7 eukaryotic genomes (*Oryza sativa*, *Paramecium tetraurelia*, *Saccharomyces cerevisiae*, *Entamoeba histolytica* and the nucleomorphs of *Guillardia theta*, *Bigelowiella natans* and *Hemiselmis andersenii*) and various MGE (plasmids and viruses). In addition to this, we used MetaGeneAnnotator [46] to predict 9,456,237 protein sequences longer than 50 amino acids, from 31,779,190 contigs/reads, issued from 236 samples of microbial metagenomics (excluding projects of viral and plasmid metagenomics, see Additional file 1: Table S1 for a detailed list).

### Selection of gene families with a strong ancient genealogical signal

We reconstructed the gene network of the initial dataset, defining significant similarity for sequences with a BLAST e-value < 1e-5 and >30 % identity, inferred as the BLAST identity multiplied by hit coverage for the shortest sequence, as in [47]. We computed the conductance [48] of each Domain in each connected component of this network, estimated by the ratio of external edges (from nodes within the Domain to nodes outside the Domain) over internal edges (between nodes within the Domain only). We found 86 connected components with a conductance lower than 0.4 for two Domains, i.e. in which sequences were clearly structured in at least 2 conserved groups corresponding to 2 Domains of Life between which there was no or little evidence of LGT in the graph. These 86 components, called nuclei hereafter, comprised 10,673 sequences. They correspond to homologous sequences, but not necessarily to orthologs.

### Rounds of BLAST searches to gather environmental homologs

We BLASTed the 10,673 nuclei sequences against the 9,456,237 predicted environmental ORFs (TBLASTN, e-value threshold = 1e-5, 500 hits). Then, the 232,660 retrieved environmental ORFs were used as seeds for a second round of BLASTN (to avoid possible detection problems caused by frameshifts, e-value threshold = 1e-5, 500 hits) against the predicted environmental ORFs to look for their own homologs in the environment. This protocol yielded a dataset with a total of 309,245 sequences (298,578 environmental ones) to reconstruct a sequence network with nuclei and environmental sequences.

### Reconstruction of exploratory similarity networks

Environmental sequences were translated into proteins, and the previous 309,245 sequences were BLASTed all against all (BLASTP, e-value threshold = 1e-5, 5000 hits to avoid artefactual increases in the distance between environmental ORFs and nuclei in similarity networks, in case a given sequence from a nuclei hits > 500 environmental ORFs). BLAST results were used to reconstruct an exploratory similarity network, where nodes represent sequences and a “homology edge” is created between two nodes if the two sequences show a BLAST e-value < 1e-5, more than 30 % identity and a match covering at least 80 % of both the compared sequences. This latter condition ensures that no two sequences are connected by the mere sharing of a gene fragment (e.g. a domain). This similarity network retained 131,162 environmental sequences, and yielded 82 connected components: adding environmental sequences made most of the nuclei grow into independant connected component, but a in a few cases two nuclei were united into the same component.

### Analyses of the exploratory similarity networks

All the environmental sequences from each of the 82 connected components were BLASTed against the July 2013 release of Genbank *nr* database (BLASTP, e-value threshold 1e-5) to quantify the identity percentage between each of these environmental ORFs and its closest sequence in *nr*, called closest published relative (CPR). Thus, comparison was made against all eukaryotic and prokaryotic sequences deposited at this date. CPR were computed relatively to the shortest sequence, as BLAST identity multiplied by hit coverage for the shortest sequence. Moreover, the topological distance of each of these environmental ORFs to a reference genome from the three Domains of life was estimated by the number of edges separating them in the graph. Class1 sequences were identified as environmental ORFs directly connected to a nucleus sequence. Class2 sequences were identified as environmental ORFs directly connected to a class1 sequence, but not to a nucleus sequence.

### Visualization of sequence spaces and landscapes

All similarity networks were displayed with Gephi v0.8.1 software (https://gephi.org), using the ‘Yifan-Hu multilevel’ layout. This particular layout ensures a maximal readability of the connected components, by placing node from densely connected parts of the network close together.

### Extraction and analysis of highly divergent closely related environmental sequences

Maximal cliques (totally connected subnetworks) of environmental sequences whose identity to a CPR was lower than 60 % were identified in the previous 82 components using MACE [49], yielding 562 subsets of closely related environmental sequences. Each of these subsets was aligned with MUSCLE v3.8 [50], then tested for potential recombination with RDP3 [39]. Corresponding nucleotide alignments were also analyzed with yn00 software from the PAML v4.7 package [51, 52]. For each clique, average dN/dS ratio was computed as the average of pairwise omega parameters. MUSCLE alignments were used to compute average residue identity, as the mean of pairwise identity percentages for each clique. All scripts used to measure conductances and network distances, and to extract largest maximal cliques in our networks are available from PL upon request.

### Phylogenetic analyses of closely related environmental sequences

For phylogenetic purposes, environmental clique amino acid sequences were aligned with their nucleus counterparts using MUSCLE, and ambiguously aligned regions were discarded with GBlocks v0.91b [40]. Only 289 streamlined alignments retained more than 10 residues, confirming that a large number of cliques contained extremely distant homologs of nucleus sequences. Unrooted maximum likelihood trees were then reconstructed from these 289 alignments using PhyML v3.0 [53] with default parameters.

## Reviewers’ comments

**Reviewer’s report 1 (Eugene Koonin, National Center for Biotechnology Information, USA)**

**Summary** : Lopez and colleagues report an extensive analysis of metagenomic sequences that demonstrates the presence of highly diverged versions of (nearly) universal genes including 18 coding for ribosomal proteins. The results are deemed compatible with the existence of "new divisions of life". I think this cautious conclusion is justified. Although some of the sequences are more distant from bacteria and archaea than the latter are from each other, a conservative standpoint is that these sequences are more likely to represent fast evolving bacteria or archaea than new domains of cellular life. Again, the authors are quite cautious in their interpretations and never overstate their case. All in all, this is an interesting and important article.

**Author’s response:** we thank the reviewer for his appreciation of our work.

**Recommandations**: I have no major recommendations. Minor recommendation: it seems desirable to cite and briefly discuss the following recent publications that are directly relevant to the theme of this article: Luef B, Frischkorn KR, Wrighton KC, Holman HY, Birarda G, Thomas BC, Singh A, Williams KH, Siegerist CE, Tringe SG, Downing KH, Comolli LR, Banfield JF. Diverse uncultivated ultra-small bacterial cells in groundwater. Nat Commun. 2015 Feb 27;6:6372 Brown CT, Hug LA, Thomas BC, Sharon I, Castelle CJ, Singh A, Wilkins MJ, Wrighton KC, Williams KH, Banfield JF. Unusual biology across a group comprising more than 15 % of domain Bacteria. Nature. 2015 Jul 9;523(7559):208–11

**Author’s response:** we agree with the reviewer and have now cited and discussed these two important articles in the introduction and conclusion.

**Minor Issues** : The text of the article requires some additional editing, preferably, by a native English speaker.

**Author’s response:** the manuscript has been edited by a native English speaker.

**Reviewer’s report 2 (William Martin, Heinrich-Heine-Universität, Germany)**

**Summary** : The authors present novel (network) methods to address diversity in envirnomental sequences. The paper is very interesting and technically an important step in the right direction to explore more of what we do not know about environmental microbial diversity. It can be published as is in my view, some language editing by a native speaker would help, I listed some but by no means all style and usage problems below.

**Author’s response:** we thank the reviewer for his comments and for pointing out the interest of the method.

**Recommandations:**

p. 1.

l. 30 life levels? reword, not clear

l 43, tres of life — > phylogenetic trees

papers should be line numbered continuously in the future, please, makes commenting easier.

life division — > divisions of life

have been constant progresses — has been constant progress

obsidian Pool , capitalization

polymorphism. 30 % of the genes — > polymorphism. Thirty percent of the genes (never start a sentence with a numeral)

Likewise, in 2005, Chouari et al. : reference number?

In 2012, Narasingarao et al. : reference number?

it seems that one could in principle come up with some novel microbial lineages, at various levels of the biological classification — > novel microbial lineages are being discovered through environmental sequencing.

microbial dark universe : was dark matter already introduced?

these data are amongst the most numerous — > these data are amongst the most abundant

seeked to identify --- sought to identify

families fulfilled these --- families fulfilling these

protocole---protocol

important amount of microbial divergent genes --- substantial number of divergent microbial genes

proved its efficiency to unravel --- was efficient in uncovering

such gene families ! --- why !

In either case, the host lineage of the divergent environmental genes would be

439 distantly related to known organismal lineages in a way that may challenge our

knowledge in evolutionary 440 microbiology. As a follow up of this proof of concept

441 analysis, it would next be interesting to perform larger comparative studies, with

442 expanded datasets, including more sequences from cultured organisms and from more

443 environments, this new dataset might identify other ancient, divergent, rarely

444 transferred between domains gene families.

Sorry, is that essential and what is it supposed to mean?

theories on the origins of eukaryotes [41] -- Woese did not revolutionize theories for eukaryote origin, he (co-)discovered archae(bacteri)a and introduced the concept of microbial phylogeny.

**Author’s response:** typos have been corrected and the manuscript has been edited in accordance with the reviewer’s points.

**Reviewer’s report 3 (James McInerney, University of Manchester, U.K.)**

**Summary** : This is an interesting paper that demonstrates quite clearly that environmental sequence space is not well known and that future work should aim to explore this. I have no real problems with the manuscript, just some minor comments.

**Author’s response:** We thank the reviewer for his interest in our work.

**Recommandations:**

Major Comments: None. Minot comments On Fig. 5, can you put a scale bar on the figures?

**Author’s response:** A scale bar has been added to the figure.

**Minor Issues**: Minor points: 1. The language needs a little work, with some of the sentences having awkward phrasing. 2. The paper is quite long. The introduction and the discussion in particular were quite long and might benefit from being reduced in length. 3. When phylogenies were made from those sequences that could be aligned, how likely is it that long-branch attraction occurred and therefore pulled the sequences out of the bacterial or archaebacterial groups?

**Author’s response:** 1) A native English speaker has edited the manuscript. 2) Introduction and discussion have been shortened so that the article is easier to read. 3) Long-branch attraction could indeed be responsible for the particular location of environmental sequences in the trees from Fig. 5. However, sequences have been trimmed by Gblocks prior to reconstruction, yielding a very small number of positions that could be reliably aligned, which should reduce (but not rule out) the likelihood of long-branch attraction. We take this opportunity to recommend again the use of networks instead of trees for exploring high sequence divergence.

## Additional files

Additional file 1: Table S1. Environmental dataset. A total of 236 environmental microbial samples was used in this study. For each sample, environmental origin, number of predicted Open Reading Frames (total and longer than 50 amino acids) and data source are given. Figure S1 Distribution of Average Nucleotide Identity of highly divergent environmental sequences. A total of 569 cliques (totally connected subnetworks) of highly divergent environmental sequences (whose identity to CPR is lower than 60 %) was identified. Sequences from each of these cliques were aligned, then their average nucleotide identity were computed, and shown in abscissa. Proportion of corresponding cliques is given in ordinate. (PDF 89 kb)
