# Supplementary material for: Highly divergent ancient gene families in metagenomic samples are compatible with additional divisions of life
Source: Biol Direct. 2015 Oct 26;10:64. doi: 10.1186/s13062-015-0092-3 (PMC4624368; doi:10.1186/s13062-015-0092-3)
Supplement: Additional file 1: Table S1. — Environmental dataset. A total of 236 environmental microbial samples was used in this study. For each sample, environmental origin, number of predicted Open Reading Frames (total and longer than 50 amino acids) and data source are given. Figure S1 Distribution of Average Nucleotide Identity of highly divergent environmental sequences. A total of 569 cliques (totally connected subnetworks) of highly divergent environmental sequences (whose identity to CPR is lower than 60 %) was identified. Sequences from each of these cliques were aligned, then their average nucleotide identity were computed, and shown in abscissa. Proportion of corresponding cliques is given in ordinate. (PDF 89 kb) [file 13062_2015_92_MOESM1_ESM.pdf]

Supplementary Material

Supplementary Table 1 : Environmental dataset. A total of 236 environmental microbial samples was used in this study. For each sample, environmental origin, number of predicted Open Reading Frames (total and longer than 50 amino acids) and data source are given.

Supplementary Figure 1: Distribution of Average Nucleotide Identity of highly divergent environmental sequences. A total of 569 cliques (totally connected subnetworks) of highly divergent environmental sequences (whose identity to CPR is lower than 60%) was identified. Sequences from each of these cliques were aligned, then their average nucleotide identity were computed, and shown in abscissa. Proportion of corresponding cliques is given in ordinate.

Filename	Environment	#ORFs	#ORFS>50	Source
AntarcticaAquatic_1.fasta	Antarctica Aquatic	116490	104903	MG RAST ID 4443683.3
AntarcticaAquatic_2.fasta	Antarctica Aquatic	10535	9632	MG RAST ID 4443680.3
AntarcticaAquatic_3.fasta	Antarctica Aquatic	9547	8875	MG RAST ID 4443679.3
AntarcticaAquatic_4.fasta	Antarctica Aquatic	44171	41965	MG RAST ID 4443681.3
AntarcticaAquatic_5.fasta	Antarctica Aquatic	282939	266909	MG RAST ID 4443682.3
AntarcticaAquatic_6.fasta	Antarctica Aquatic	280580	264549	MG RAST ID 4443684.3
AntarcticaAquatic_7.fasta	Antarctica Aquatic	28039	26396	MG RAST ID 4443685.3
AntarcticaAquatic_8.fasta	Antarctica Aquatic	94828	89432	MG RAST ID 4443686.3
AntarcticaAquatic_9.fasta	Antarctica Aquatic	92761	87149	MG RAST ID 4443687.3
Site227_0_1um.fasta	Antarctica Aquatic	107770	84195	https://portal.camera.calit2.net/gridsphere/
Site227_0_8um.fasta	Antarctica Aquatic	98499	80718	https://portal.camera.calit2.net/gridsphere/
Site227_3_0um.fasta	Antarctica Aquatic	41785	33819	https://portal.camera.calit2.net/gridsphere/
Site228_0_1um.fasta	Antarctica Aquatic	90982	68808	https://portal.camera.calit2.net/gridsphere/
Site228_0_8um.fasta	Antarctica Aquatic	86160	69758	https://portal.camera.calit2.net/gridsphere/
Site228_3_0um.fasta	Antarctica Aquatic	44139	36294	https://portal.camera.calit2.net/gridsphere/
Site229_0_1um.fasta	Antarctica Aquatic	92987	71783	https://portal.camera.calit2.net/gridsphere/
Site229_0_8um.fasta	Antarctica Aquatic	59663	47312	https://portal.camera.calit2.net/gridsphere/
Site229_3_0um.fasta	Antarctica Aquatic	43132	35288	https://portal.camera.calit2.net/gridsphere/
Site230_0_1um.fasta	Antarctica Aquatic	72760	60393	https://portal.camera.calit2.net/gridsphere/
Site230_0_8um.fasta	Antarctica Aquatic	83496	67983	https://portal.camera.calit2.net/gridsphere/
BBAY01.fasta	Botany Bay	89204	84671	MG RAST ID 4443688.3
BBAY02.fasta	Botany Bay	11537	10624	MG RAST ID 4443689.3
BBAY04.fasta	Botany Bay	7605	6610	MG RAST ID 4443691.3
BBAY14.fasta	Botany Bay	1331	897	MG RAST ID 4443688.6
BBAY15.fasta	Botany Bay	116594	110056	MG RAST ID 4443693.3
ChickenCecumA.fasta	Chicken Cecum A	6	1	MG RAST ID 4440283.3
ChickenCecumB.fasta	Chicken Cecum B	7	0	MG RAST ID 4440284.3
GlacierIceMetagenome_SRX000607.fasta	Glacier	17290	12163	http://www.ncbi.nlm.nih.gov/pubmed?term=19801459
4440055.3.fasta	Healthy gut bacteria	0	1	http://www.theseed.org/DinsdaleSupplementalMaterial/
4440411.3.fasta	Healthy Prebead tank microbes	1	0	http://www.theseed.org/DinsdaleSupplementalMaterial/
4440059.3.fasta	Healthy slime bacteria	0	0	http://www.theseed.org/DinsdaleSupplementalMaterial/
4440413.3.fasta	Healthy Tilapia pond microbes	1	0	http://www.theseed.org/DinsdaleSupplementalMaterial/
4440419.3.fasta	High saltern microbial (HB1128)	0	0	http://www.theseed.org/DinsdaleSupplementalMaterial/
4440061.3.fasta	Highborne Cay stromatolite bacteria	16	0	http://www.theseed.org/DinsdaleSupplementalMaterial/
BATS216_20m_SG.fasta	Hot Aloha	108483	101423	http://www.ncbi.nlm.nih.gov/sites/entrez/16439655
BATS216_50m_SG.fasta	Hot Aloha	105004	97772	http://www.ncbi.nlm.nih.gov/sites/entrez/16439655
HOT179_125m_cDNA.fasta	Hot Aloha	8	1	http://www.ncbi.nlm.nih.gov/sites/entrez/16439655
HOT179_125m_gDNA.fasta	Hot Aloha	48	0	http://www.ncbi.nlm.nih.gov/sites/entrez/16439655
HOT179_125m_SG.fasta	Hot Aloha	95854	89065	http://www.ncbi.nlm.nih.gov/sites/entrez/16439655
HOT179_25m_SG.fasta	Hot Aloha	100844	92542	http://www.ncbi.nlm.nih.gov/sites/entrez/16439655
JGI_SMPL_HF10_10-07-02.fasta	Hot Aloha	4895	4562	http://www.ncbi.nlm.nih.gov/sites/entrez/16439655
JGI_SMPL_HF130_10-06-02.fasta	Hot Aloha	3429	3199	http://www.ncbi.nlm.nih.gov/sites/entrez/16439655
JGI_SMPL_HF4000_12-21-03.fasta	Hot Aloha	7396	7011	http://www.ncbi.nlm.nih.gov/sites/entrez/16439655
JGI_SMPL_HF500_10-06-02.fasta	Hot Aloha	5771	5409	http://www.ncbi.nlm.nih.gov/sites/entrez/16439655
JGI_SMPL_HF70_10-07-02.fasta	Hot Aloha	6834	6241	http://www.ncbi.nlm.nih.gov/sites/entrez/16439655
BGI_GeneSet20090523.fasta	Human microbiome Qin2010	1529492	1522788	http:///www.bork.embl.de/~arumugam/Qin_et_al_2010/
MH0001.seq.fasta	Human microbiome Qin2010	9705	9533	http:///www.bork.embl.de/~arumugam/Qin_et_al_2010/
MH0002.seq.fasta	Human microbiome Qin2010	44320	43499	http:///www.bork.embl.de/~arumugam/Qin_et_al_2010/
MH0003.seq.fasta	Human microbiome Qin2010	64601	63771	http:///www.bork.embl.de/~arumugam/Qin_et_al_2010/
MH0004.seq.fasta	Human microbiome Qin2010	14479	14281	http:///www.bork.embl.de/~arumugam/Qin_et_al_2010/
MH0005.seq.fasta	Human microbiome Qin2010	9064	8929	http:///www.bork.embl.de/~arumugam/Qin_et_al_2010/
MH0006.seq.fasta	Human microbiome Qin2010	100175	98429	http:///www.bork.embl.de/~arumugam/Qin_et_al_2010/
MH0007.seq.fasta	Human microbiome Qin2010	13227	12902	http:///www.bork.embl.de/~arumugam/Qin_et_al_2010/
MH0008.seq.fasta	Human microbiome Qin2010	18064	17786	http:///www.bork.embl.de/~arumugam/Qin_et_al_2010/
MH0009.seq.fasta	Human microbiome Qin2010	62530	61529	http:///www.bork.embl.de/~arumugam/Qin_et_al_2010/
MH0010.seq.fasta	Human microbiome Qin2010	16233	15984	http:///www.bork.embl.de/~arumugam/Qin_et_al_2010/
MH0011.seq.fasta	Human microbiome Qin2010	71135	70024	http:///www.bork.embl.de/~arumugam/Qin_et_al_2010/
MH0012.seq.fasta	Human microbiome Qin2010	132734	130930	http:///www.bork.embl.de/~arumugam/Qin_et_al_2010/
MH0013.seq.fasta	Human microbiome Qin2010	17551	17250	http:///www.bork.embl.de/~arumugam/Qin_et_al_2010/
MH0014.seq.fasta	Human microbiome Qin2010	68504	67622	http:///www.bork.embl.de/~arumugam/Qin_et_al_2010/
MH0015.seq.fasta	Human microbiome Qin2010	15686	15449	http:///www.bork.embl.de/~arumugam/Qin_et_al_2010/
MH0016.seq.fasta	Human microbiome Qin2010	62071	61212	http:///www.bork.embl.de/~arumugam/Qin_et_al_2010/
MH0017.seq.fasta	Human microbiome Qin2010	15527	15304	http:///www.bork.embl.de/~arumugam/Qin_et_al_2010/
MH0018.seq.fasta	Human microbiome Qin2010	7900	7753	http:///www.bork.embl.de/~arumugam/Qin_et_al_2010/
MH0019.seq.fasta	Human microbiome Qin2010	20115	19862	http:///www.bork.embl.de/~arumugam/Qin_et_al_2010/
MH0020.seq.fasta	Human microbiome Qin2010	58224	57572	http:///www.bork.embl.de/~arumugam/Qin_et_al_2010/
MH0021.seq.fasta	Human microbiome Qin2010	31315	30692	http:///www.bork.embl.de/~arumugam/Qin_et_al_2010/
MH0022.seq.fasta	Human microbiome Qin2010	13728	13555	http:///www.bork.embl.de/~arumugam/Qin_et_al_2010/
MH0023.seq.fasta	Human microbiome Qin2010	13938	13682	http:///www.bork.embl.de/~arumugam/Qin_et_al_2010/
MH0024.seq.fasta	Human microbiome Qin2010	24189	23767	http:///www.bork.embl.de/~arumugam/Qin_et_al_2010/
MH0025.seq.fasta	Human microbiome Qin2010	56058	55240	http:///www.bork.embl.de/~arumugam/Qin_et_al_2010/
MH0026.seq.fasta	Human microbiome Qin2010	41939	41397	http:///www.bork.embl.de/~arumugam/Qin_et_al_2010/
MH0027.seq.fasta	Human microbiome Qin2010	6413	6243	http:///www.bork.embl.de/~arumugam/Qin_et_al_2010/
MH0028.seq.fasta	Human microbiome Qin2010	57039	56252	http:///www.bork.embl.de/~arumugam/Qin_et_al_2010/
MH0030.seq.fasta	Human microbiome Qin2010	38721	38115	http:///www.bork.embl.de/~arumugam/Qin_et_al_2010/
MH0031.seq.fasta	Human microbiome Qin2010	38928	38135	http:///www.bork.embl.de/~arumugam/Qin_et_al_2010/
MH0032.seq.fasta	Human microbiome Qin2010	27622	27100	http:///www.bork.embl.de/~arumugam/Qin_et_al_2010/
MH0033.seq.fasta	Human microbiome Qin2010	56244	55389	http:///www.bork.embl.de/~arumugam/Qin_et_al_2010/
MH0034.seq.fasta	Human microbiome Qin2010	19967	19596	http:///www.bork.embl.de/~arumugam/Qin_et_al_2010/
MH0035.seq.fasta	Human microbiome Qin2010	51823	50897	http:///www.bork.embl.de/~arumugam/Qin_et_al_2010/
MH0036.seq.fasta	Human microbiome Qin2010	48730	48001	http:///www.bork.embl.de/~arumugam/Qin_et_al_2010/
MH0037.seq.fasta	Human microbiome Qin2010	43533	42915	http:///www.bork.embl.de/~arumugam/Qin_et_al_2010/
MH0038.seq.fasta	Human microbiome Qin2010	49904	49252	http:///www.bork.embl.de/~arumugam/Qin_et_al_2010/
MH0039.seq.fasta	Human microbiome Qin2010	52294	51348	http:///www.bork.embl.de/~arumugam/Qin_et_al_2010/
MH0040.seq.fasta	Human microbiome Qin2010	59139	58292	http:///www.bork.embl.de/~arumugam/Qin_et_al_2010/
MH0041.seq.fasta	Human microbiome Qin2010	49385	48631	http:///www.bork.embl.de/~arumugam/Qin_et_al_2010/
MH0042.seq.fasta	Human microbiome Qin2010	47677	46899	http:///www.bork.embl.de/~arumugam/Qin_et_al_2010/
MH0043.seq.fasta	Human microbiome Qin2010	59344	57951	http:///www.bork.embl.de/~arumugam/Qin_et_al_2010/
MH0044.seq.fasta	Human microbiome Qin2010	56873	56065	http:///www.bork.embl.de/~arumugam/Qin_et_al_2010/
MH0045.seq.fasta	Human microbiome Qin2010	46126	45316	http:///www.bork.embl.de/~arumugam/Qin_et_al_2010/
MH0046.seq.fasta	Human microbiome Qin2010	41238	40594	http:///www.bork.embl.de/~arumugam/Qin_et_al_2010/
MH0047.seq.fasta	Human microbiome Qin2010	18183	17849	http:///www.bork.embl.de/~arumugam/Qin_et_al_2010/
MH0048.seq.fasta	Human microbiome Qin2010	26290	25732	http:///www.bork.embl.de/~arumugam/Qin_et_al_2010/
MH0049.seq.fasta	Human microbiome Qin2010	25082	24696	http:///www.bork.embl.de/~arumugam/Qin_et_al_2010/
MH0050.seq.fasta	Human microbiome Qin2010	55374	54459	http:///www.bork.embl.de/~arumugam/Qin_et_al_2010/
MH0051.seq.fasta	Human microbiome Qin2010	28884	28289	http:///www.bork.embl.de/~arumugam/Qin_et_al_2010/
MH0052.seq.fasta	Human microbiome Qin2010	32570	31926	http:///www.bork.embl.de/~arumugam/Qin_et_al_2010/
MH0053.seq.fasta	Human microbiome Qin2010	48379	47704	http:///www.bork.embl.de/~arumugam/Qin_et_al_2010/
MH0054.seq.fasta	Human microbiome Qin2010	56449	55737	http:///www.bork.embl.de/~arumugam/Qin_et_al_2010/
MH0055.seq.fasta	Human microbiome Qin2010	56526	55737	http:///www.bork.embl.de/~arumugam/Qin_et_al_2010/
MH0056.seq.fasta	Human microbiome Qin2010	44156	43388	http:///www.bork.embl.de/~arumugam/Qin_et_al_2010/
MH0057.seq.fasta	Human microbiome Qin2010	38384	37900	http:///www.bork.embl.de/~arumugam/Qin_et_al_2010/
MH0058.seq.fasta	Human microbiome Qin2010	58074	57099	http:///www.bork.embl.de/~arumugam/Qin_et_al_2010/
MH0059.seq.fasta	Human microbiome Qin2010	63906	62895	http:///www.bork.embl.de/~arumugam/Qin_et_al_2010/
MH0060.seq.fasta	Human microbiome Qin2010	46461	45824	http:///www.bork.embl.de/~arumugam/Qin_et_al_2010/
MH0061.seq.fasta	Human microbiome Qin2010	43172	42596	http:///www.bork.embl.de/~arumugam/Qin_et_al_2010/
MH0062.seq.fasta	Human microbiome Qin2010	46012	45325	http:///www.bork.embl.de/~arumugam/Qin_et_al_2010/
MH0063.seq.fasta	Human microbiome Qin2010	46152	45339	http:///www.bork.embl.de/~arumugam/Qin_et_al_2010/
MH0064.seq.fasta	Human microbiome Qin2010	45251	44502	http:///www.bork.embl.de/~arumugam/Qin_et_al_2010/
MH0065.seq.fasta	Human microbiome Qin2010	56359	55320	http:///www.bork.embl.de/~arumugam/Qin_et_al_2010/
MH0066.seq.fasta	Human microbiome Qin2010	50140	49495	http:///www.bork.embl.de/~arumugam/Qin_et_al_2010/
MH0067.seq.fasta	Human microbiome Qin2010	58076	57420	http:///www.bork.embl.de/~arumugam/Qin_et_al_2010/
MH0068.seq.fasta	Human microbiome Qin2010	47259	46673	http:///www.bork.embl.de/~arumugam/Qin_et_al_2010/
MH0069.seq.fasta	Human microbiome Qin2010	60995	59910	http:///www.bork.embl.de/~arumugam/Qin_et_al_2010/
MH0070.seq.fasta	Human microbiome Qin2010	60653	59545	http:///www.bork.embl.de/~arumugam/Qin_et_al_2010/
MH0071.seq.fasta	Human microbiome Qin2010	51469	50739	http:///www.bork.embl.de/~arumugam/Qin_et_al_2010/

MH0072.seq.fasta	Human microbiome Qin2010	36370	35662	http://www.bork.embl.de/~arumugam/Qin_et_al_2010/
MH0073.seq.fasta	Human microbiome Qin2010	43953	43219	http://www.bork.embl.de/~arumugam/Qin_et_al_2010/
MH0074.seq.fasta	Human microbiome Qin2010	57619	56976	http://www.bork.embl.de/~arumugam/Qin_et_al_2010/
MH0075.seq.fasta	Human microbiome Qin2010	53166	52311	http://www.bork.embl.de/~arumugam/Qin_et_al_2010/
MH0076.seq.fasta	Human microbiome Qin2010	39036	38281	http://www.bork.embl.de/~arumugam/Qin_et_al_2010/
MH0077.seq.fasta	Human microbiome Qin2010	54384	53366	http://www.bork.embl.de/~arumugam/Qin_et_al_2010/
MH0078.seq.fasta	Human microbiome Qin2010	15078	14700	http://www.bork.embl.de/~arumugam/Qin_et_al_2010/
MH0079.seq.fasta	Human microbiome Qin2010	31877	31286	http://www.bork.embl.de/~arumugam/Qin_et_al_2010/
MH0080.seq.fasta	Human microbiome Qin2010	58876	57787	http://www.bork.embl.de/~arumugam/Qin_et_al_2010/
MH0081.seq.fasta	Human microbiome Qin2010	56013	55022	http://www.bork.embl.de/~arumugam/Qin_et_al_2010/
MH0082.seq.fasta	Human microbiome Qin2010	61842	60761	http://www.bork.embl.de/~arumugam/Qin_et_al_2010/
MH0083.seq.fasta	Human microbiome Qin2010	57445	56657	http://www.bork.embl.de/~arumugam/Qin_et_al_2010/
MH0084.seq.fasta	Human microbiome Qin2010	25963	25593	http://www.bork.embl.de/~arumugam/Qin_et_al_2010/
MH0085.seq.fasta	Human microbiome Qin2010	45650	45039	http://www.bork.embl.de/~arumugam/Qin_et_al_2010/
MH0086.seq.fasta	Human microbiome Qin2010	80256	79130	http://www.bork.embl.de/~arumugam/Qin_et_al_2010/
O2.UC-1.seq.fasta	Human microbiome Qin2010	34915	34310	http://www.bork.embl.de/~arumugam/Qin_et_al_2010/
O2.UC-11.seq.fasta	Human microbiome Qin2010	26276	25651	http://www.bork.embl.de/~arumugam/Qin_et_al_2010/
O2.UC-12.seq.fasta	Human microbiome Qin2010	41536	40802	http://www.bork.embl.de/~arumugam/Qin_et_al_2010/
O2.UC-13.seq.fasta	Human microbiome Qin2010	54232	53346	http://www.bork.embl.de/~arumugam/Qin_et_al_2010/
O2.UC-14.seq.fasta	Human microbiome Qin2010	22202	21763	http://www.bork.embl.de/~arumugam/Qin_et_al_2010/
O2.UC-16.seq.fasta	Human microbiome Qin2010	16427	16037	http://www.bork.embl.de/~arumugam/Qin_et_al_2010/
O2.UC-17.seq.fasta	Human microbiome Qin2010	19239	18886	http://www.bork.embl.de/~arumugam/Qin_et_al_2010/
O2.UC-18.seq.fasta	Human microbiome Qin2010	44325	43853	http://www.bork.embl.de/~arumugam/Qin_et_al_2010/
O2.UC-19.seq.fasta	Human microbiome Qin2010	47826	47108	http://www.bork.embl.de/~arumugam/Qin_et_al_2010/
O2.UC-20.seq.fasta	Human microbiome Qin2010	40938	40247	http://www.bork.embl.de/~arumugam/Qin_et_al_2010/
O2.UC-21.seq.fasta	Human microbiome Qin2010	20628	20236	http://www.bork.embl.de/~arumugam/Qin_et_al_2010/
O2.UC-22.seq.fasta	Human microbiome Qin2010	49015	48251	http://www.bork.embl.de/~arumugam/Qin_et_al_2010/
O2.UC-23.seq.fasta	Human microbiome Qin2010	32607	32151	http://www.bork.embl.de/~arumugam/Qin_et_al_2010/
O2.UC-24.seq.fasta	Human microbiome Qin2010	40718	40103	http://www.bork.embl.de/~arumugam/Qin_et_al_2010/
O2.UC-4.seq.fasta	Human microbiome Qin2010	45174	44394	http://www.bork.embl.de/~arumugam/Qin_et_al_2010/
V1.CD-1.seq.fasta	Human microbiome Qin2010	29601	29146	http://www.bork.embl.de/~arumugam/Qin_et_al_2010/
V1.CD-11.seq.fasta	Human microbiome Qin2010	50757	50062	http://www.bork.embl.de/~arumugam/Qin_et_al_2010/
V1.CD-12.seq.fasta	Human microbiome Qin2010	29767	29272	http://www.bork.embl.de/~arumugam/Qin_et_al_2010/
V1.CD-13.seq.fasta	Human microbiome Qin2010	54407	53533	http://www.bork.embl.de/~arumugam/Qin_et_al_2010/
V1.CD-14.seq.fasta	Human microbiome Qin2010	60291	59339	http://www.bork.embl.de/~arumugam/Qin_et_al_2010/
V1.CD-15.seq.fasta	Human microbiome Qin2010	30238	29885	http://www.bork.embl.de/~arumugam/Qin_et_al_2010/
V1.CD-2.seq.fasta	Human microbiome Qin2010	8200	7417	http://www.bork.embl.de/~arumugam/Qin_et_al_2010/
V1.CD-3.seq.fasta	Human microbiome Qin2010	37699	37172	http://www.bork.embl.de/~arumugam/Qin_et_al_2010/
V1.CD-4.seq.fasta	Human microbiome Qin2010	52488	51632	http://www.bork.embl.de/~arumugam/Qin_et_al_2010/
V1.CD-6.seq.fasta	Human microbiome Qin2010	37845	37236	http://www.bork.embl.de/~arumugam/Qin_et_al_2010/
V1.CD-8.seq.fasta	Human microbiome Qin2010	60741	59778	http://www.bork.embl.de/~arumugam/Qin_et_al_2010/
V1.CD-9.seq.fasta	Human microbiome Qin2010	57918	57191	http://www.bork.embl.de/~arumugam/Qin_et_al_2010/
V1.UC-10.seq.fasta	Human microbiome Qin2010	59332	58430	http://www.bork.embl.de/~arumugam/Qin_et_al_2010/
V1.UC-13.seq.fasta	Human microbiome Qin2010	49182	48634	http://www.bork.embl.de/~arumugam/Qin_et_al_2010/
V1.UC-14.seq.fasta	Human microbiome Qin2010	54997	54081	http://www.bork.embl.de/~arumugam/Qin_et_al_2010/
V1.UC-15.seq.fasta	Human microbiome Qin2010	57413	56649	http://www.bork.embl.de/~arumugam/Qin_et_al_2010/
V1.UC-17.seq.fasta	Human microbiome Qin2010	41186	40548	http://www.bork.embl.de/~arumugam/Qin_et_al_2010/
V1.UC-18.seq.fasta	Human microbiome Qin2010	50305	49678	http://www.bork.embl.de/~arumugam/Qin_et_al_2010/
V1.UC-19.seq.fasta	Human microbiome Qin2010	64271	63314	http://www.bork.embl.de/~arumugam/Qin_et_al_2010/
V1.UC-21.seq.fasta	Human microbiome Qin2010	47694	46908	http://www.bork.embl.de/~arumugam/Qin_et_al_2010/
V1.UC-6.seq.fasta	Human microbiome Qin2010	58897	57916	http://www.bork.embl.de/~arumugam/Qin_et_al_2010/
V1.UC-7.seq.fasta	Human microbiome Qin2010	28217	27872	http://www.bork.embl.de/~arumugam/Qin_et_al_2010/
V1.UC-8.seq.fasta	Human microbiome Qin2010	65498	64408	http://www.bork.embl.de/~arumugam/Qin_et_al_2010/
V1.UC-9.seq.fasta	Human microbiome Qin2010	50267	49485	http://www.bork.embl.de/~arumugam/Qin_et_al_2010/
F1S.fasta	Japanese Microbiome	26089	24920	http://www.ncbi.nlm.nih.gov/pubmed/17916580
F1T.fasta	Japanese Microbiome	26164	24971	http://www.ncbi.nlm.nih.gov/pubmed/17916580
F1U.fasta	Japanese Microbiome	18319	17476	http://www.ncbi.nlm.nih.gov/pubmed/17916580
F2V.fasta	Japanese Microbiome	29005	27659	http://www.ncbi.nlm.nih.gov/pubmed/17916580
F2W.fasta	Japanese Microbiome	27387	26191	http://www.ncbi.nlm.nih.gov/pubmed/17916580
F2X.fasta	Japanese Microbiome	25712	24569	http://www.ncbi.nlm.nih.gov/pubmed/17916580
F2Y.fasta	Japanese Microbiome	29424	28194	http://www.ncbi.nlm.nih.gov/pubmed/17916580
InA.fasta	Japanese Microbiome	20271	19417	http://www.ncbi.nlm.nih.gov/pubmed/17916580
InB.fasta	Japanese Microbiome	9887	9528	http://www.ncbi.nlm.nih.gov/pubmed/17916580
InD.fasta	Japanese Microbiome	31760	30268	http://www.ncbi.nlm.nih.gov/pubmed/17916580
InE.fasta	Japanese Microbiome	18796	17871	http://www.ncbi.nlm.nih.gov/pubmed/17916580
InM.fasta	Japanese Microbiome	19268	18619	http://www.ncbi.nlm.nih.gov/pubmed/17916580
InR.fasta	Japanese Microbiome	29183	27723	http://www.ncbi.nlm.nih.gov/pubmed/17916580
4440463.3.fasta	Lean Mice	4014	3797	Turnbaugh et al., Nature. 2006 444(7122):1027-31
4440041.3.fasta	Line Islands Christmas Reef B3 bacteria	5	2	Dinsdale et al, PLoS One, 3:e1584
4440037.3.fasta	Line Islands Kingman Reef B2 bacteria	3	0	Dinsdale et al, PLoS One, 3:e1584
4440039.3.fasta	Line Islands Palmyra F8 Bacteria	15	0	Dinsdale et al, PLoS One, 3:e1584
4440279.3.fasta	Line Islands Tabuaeran B1 Bacteria	3	0	Dinsdale et al, PLoS One, 3:e1584
4440426.3.fasta	Low salinity microbial (LB1128)	3	0	http://www.theseed.org/DinsdaleSupplementalMaterial/
4440437.3.fasta	Low saltern microbes (Pond 11)	7	0	http://www.theseed.org/DinsdaleSupplementalMaterial/
4440425.3.fasta	Medium salinity microbial (MB1116)	0	0	http://www.theseed.org/DinsdaleSupplementalMaterial/
4440435.3.fasta	Medium saltern microbes (MB1110)	1	0	http://www.theseed.org/DinsdaleSupplementalMaterial/
4440434.3.fasta	Medium saltern microbes (MB1111)	0	0	http://www.theseed.org/DinsdaleSupplementalMaterial/
4440416.3.fasta	Medium saltern microbial (MB1128)	0	0	http://www.theseed.org/DinsdaleSupplementalMaterial/
mb2000jd298_1.fasta	Monterey Bay	7707	5069	MG RAST ID 4443713.3
mb2000jd298_2.fasta	Monterey Bay	6667	4419	MG RAST ID 4443712.3
mb2001jd115_1.fasta	Monterey Bay	5145	3456	MG RAST ID 4443714.3
mb2001jd115_2.fasta	Monterey Bay	4909	3211	MG RAST ID 4443715.3
mb2001jd135_1.fasta	Monterey Bay	6658	4435	MG RAST ID 4443716.3
mb2001jd135_2.fasta	Monterey Bay	5596	3662	MG RAST ID 4443717.3
4440056.3.fasta	Morbid gut bacteria	1	1	MG RAST ID 4440056.3
4440066.3.fasta	Morbid slime bacteria	2	0	MG RAST ID 4440066.3
4440464.3.fasta	Obese Mice	4841	4490	Turnbaugh et al., Nature. 2006 444(7122):1027-31
NCBI_projectID_13729.fasta	Pacific Beach	4480	4339	http://genomesonline.org/cgi-bin/GOLD/bin/GOLDCards.cgi?goldstamp=Gm00026
4440319.3.fasta	Porites astreoides microbial extraction	13	0	Wegley, et al Environ Microbiol. 2007 9(11):2707-19.
4440378.3.fasta	Porites compressa control treated bacteria	8	0	http://www.ncbi.nlm.nih.gov/pubmed/19397678
4440372.3.fasta	Porites compressa DOC treated microbes	2	0	http://www.ncbi.nlm.nih.gov/pubmed/19397678
4440381.3.fasta	Porites compressa nutrient treated microbes	3	0	http://www.ncbi.nlm.nih.gov/pubmed/19397678
4440379.3.fasta	Porites compressa pH treated microbes	3	0	http://www.ncbi.nlm.nih.gov/pubmed/19397678
4440373.3.fasta	Porites compressa temperature treated bacteria	3	0	http://www.ncbi.nlm.nih.gov/pubmed/19397678
4440380.3.fasta	Porites compressa time zero bacteria	2	0	http://www.ncbi.nlm.nih.gov/pubmed/19397678
4440067.3.fasta	Pozas Azule II stromatolite microbes	12	0	http://www.theseed.org/DinsdaleSupplementalMaterial/
4440060.3.fasta	Rios Mesquites Stromatolites bacteria	64	1	http://www.theseed.org/DinsdaleSupplementalMaterial/
4440329.3.fasta	Salton Sea Bacteria 1	5	0	MG RAST ID 4440329
FN56_all.fasta	Sapelo Island	11286	6185	http://www.ncbi.nlm.nih.gov/pubmed/20844569
FN57_all.fasta	Sapelo Island	10427	5705	http://www.ncbi.nlm.nih.gov/pubmed/20844569
4440282.3.fasta	Soudan Black Stuff	14	0	http://www.theseed.org/DinsdaleSupplementalMaterial/
4440281.3.fasta	Soudan Red Stuff	30	3	http://www.theseed.org/DinsdaleSupplementalMaterial/
BC3.fasta	Syntrophic Partners	2217	1493	https://portal.camera.calit2.net/gridsphere/
MDA.fasta	Syntrophic Partners	2404	1941	https://portal.camera.calit2.net/gridsphere/
4440440.3.fasta	Tilapia pond microbes	23	0	http://www.theseed.org/DinsdaleSupplementalMaterial/
4440355.3.fasta	Total microbial community from cow rumens	7	0	http://www.theseed.org/DinsdaleSupplementalMaterial/
4440356.3.fasta	Total microbial community from cow rumens	3	1	http://www.theseed.org/DinsdaleSupplementalMaterial/
4440357.3.fasta	Total microbial community from cow rumens	7	0	http://www.theseed.org/DinsdaleSupplementalMaterial/
4440387.3.fasta	Total microbial community from cow rumens	8	0	http://www.theseed.org/DinsdaleSupplementalMaterial/
4440360.3.fasta	Total microbial community from DMSP treated seawater	1	0	http://www.theseed.org/DinsdaleSupplementalMaterial/
4440364.3.fasta	Total microbial community from DMSP treated seawater	1	0	http://www.theseed.org/DinsdaleSupplementalMaterial/
4440363.3.fasta	Total microbial community from Vanillate treated seawater	2	0	http://www.theseed.org/DinsdaleSupplementalMaterial/
4440365.3.fasta	Total microbial community from Vanillate treated seawater	0	0	http://www.theseed.org/DinsdaleSupplementalMaterial/
4440422.3.fasta	Tpond microbe 3	2	0	http://www.theseed.org/DinsdaleSupplementalMaterial/
formaldehyde_fas	Washington Lake	16013	15373	http://genome.jgi-psf.org/lakwa/lakwa.info.html
formate_fas	Washington Lake	4699	4622	http://genome.jgi-psf.org/lakwa/lakwa.info.html
methane_fas	Washington Lake	17051	16542	http://genome.jgi-psf.org/lakwa/lakwa.info.html

methanol_fas	Washington Lake	16461	16016	http://genome.jgi-psf.org/lakwa/lakwa.info.html
methylamine_fas	Washington Lake	14871	14550	http://genome.jgi-psf.org/lakwa/lakwa.info.html
AerobicBasingenomic.fasta	Waste Water Mallard	6386	4763	http://sra.dnanexus.com/runs/SRR001308

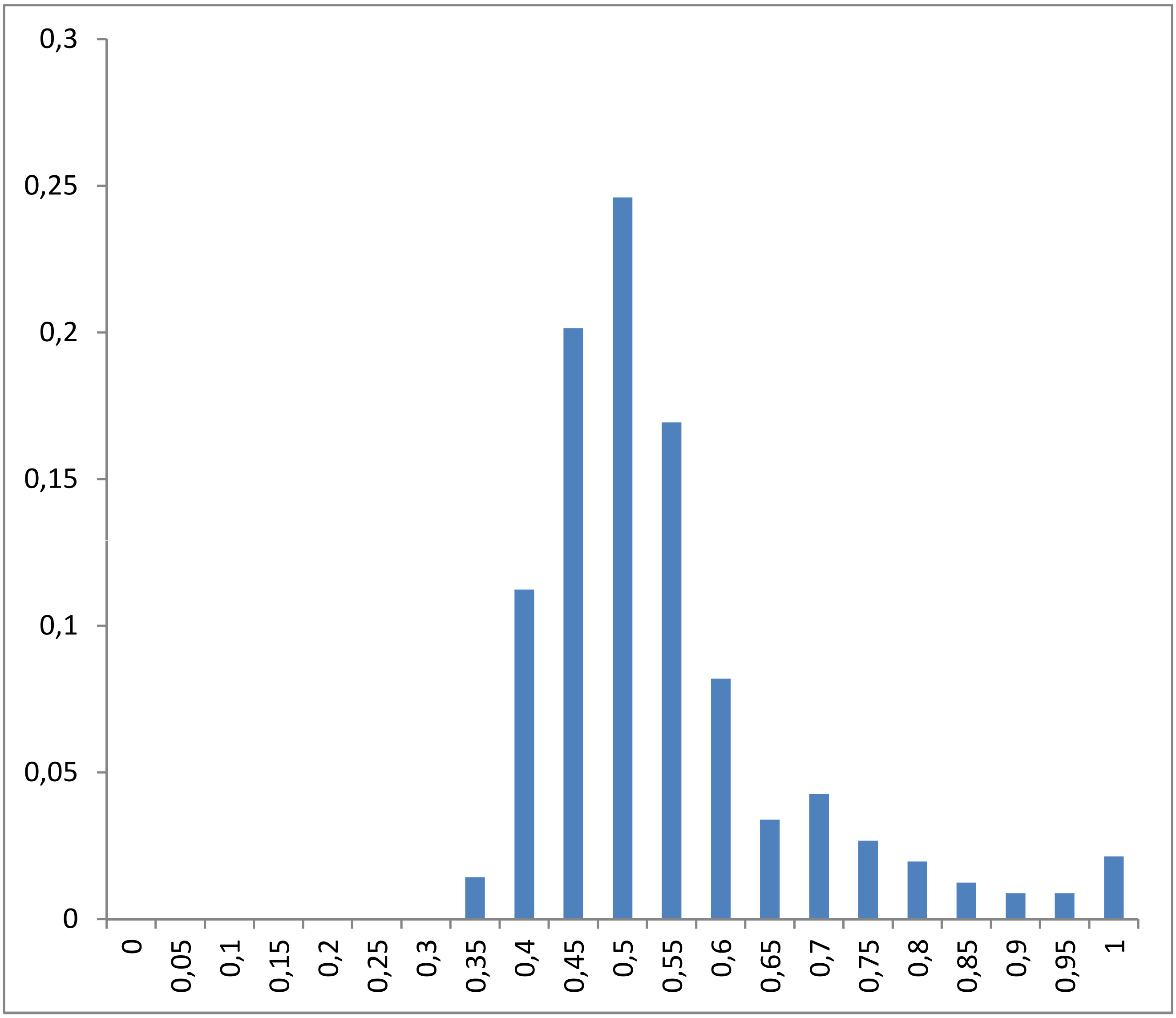
0,3
0,25
0,2
0,15
0,1
0,05
0
0
0,05
0,1
0,15
0,2
0,25
0,3
0,35
0,4
0,45
0,5
0,55
0,6
0,65
0,7
0,75
0,8
0,85
0,9
0,95
1
